# Multi-enzyme supplementation enhances performance, intestinal and skeletal development in broilers fed low-nutrient diets

**DOI:** 10.1016/j.psj.2025.106234

**Published:** 2025-12-10

**Authors:** Yuanli Cai, Mingchen Zhang, Okasha Hamada, Zhigang Song

**Affiliations:** a College of Life Science, Qilu Normal University, Jinan, Shandong 250200, China; b Key Laboratory of Efficient Utilization of Non-Grain Feed Resources; Co-construction by Ministry and Province), Ministry of Agriculture and Rural Affairs, Shandong Provincial Key Laboratory of Animal Nutrition and Efficient Feeding, Department of Animal Science, Shandong Agricultural University, Taian, Shandong 271017, China; c Animal Production Department, Faculty of Agriculture, Benha University, Moshtohor 13736, Egypt

**Keywords:** Broiler chicken, Multi-enzyme, Tibial development, Digestive enzyme activity

## Abstract

This study investigated the effects of multi-enzyme supplementation on broilers fed a zeolite-diluted, low-nutrient diet, focusing on growth performance, digestive physiology, and tibial development. A total of 384 one-day-old Arbor Acres broilers were randomly allocated to a 2 × 2 factorial arrangement, receiving either a basal diet or a low-nutrient diet (reduced in metabolic energy, crude protein, calcium, and phosphorus), each without or with a multi-enzyme preparation. The results showed that enzyme supplementation significantly improved growth performance, including body weight (day 21 and day 35), feed intake (day 21) and the feed efficiency. While the zeolite-diluted, low-nutrient diet stimulated the relative lengthening of the small intestine at 21 days, enzyme addition normalized this morphology and significantly enhanced duodenal amylase activity. Furthermore, enzyme supplementation effectively counteracted the impaired tibial development and reduced calcium content induced by the zeolite-diluted, low-nutrient diet. The study concluded that the multi-enzyme supplementation effectively mitigated the adverse effects of nutrient reduction by improving growth performance, digestive function, and skeletal development in broilers.

## Introduction

The poultry industry plays a critical role in meeting the global demand for animal protein, with broiler chickens being one of the most efficient converters of feed into meat. However, the reliance on plant-based feed ingredients, such as corn, soybean meal, and wheat, introduces challenges due to the presence of anti-nutritional factors (ANFs), including phytic acid and non-starch polysaccharides (NSP) (Meng and Slominski, 2005). Phytic acid binds essential minerals such as phosphorus and calcium, reducing their bioavailability, while NSP increases digesta viscosity, impairing nutrient digestion and absorption (Morgan et al., 2022). These limitations not only compromise the productive performance of broilers but also increase feed costs, which account for approximately 60-70 % of total production expenses (Attia et al., 2022).

The poultry industry in many countries faces challenges due to the high cost of conventional protein and energy sources (Ahiwe et al., 2022). Consequently, researchers are motivated to lower feed costs by incorporating non-conventional, cost-effective ingredients or reducing the inclusion rates of energy-dense components (Donohue and Cunningham, 2009). In poultry nutrition utilizing cereal grains and their co-products, enzyme supplementation can significantly improve dietary nutrient bioavailability (Woyengo and Nyachoti, 2011). For instance, NSP-degrading enzymes reduce gut viscosity, enhancing the digestibility of energy and amino acids (Yuan et al., 2017). Similarly, administration of phytase at elevated doses (2,500 FYT/kg) enhanced bone mineralization metrics, as evidenced by increased tibia and toe ash percentages at day 21, and the calcium content in the tibia at day 42 (Sens et al., 2021). Therefore, the inclusion of enzymes in the diet enables a reduction in feed nutrient content without compromising animal growth. However, most previous studies have focused on adding enzyme preparations to reduced-energy diets. Zhou et al. (2009) showed that adding a complex enzyme preparation (containing xylanase, α-amylase, and protease) to a low-metabolic energy (ME) diet increased the utilization of ME in broiler chickens. Conversely, another study found that incorporating enzymes into a low-ME diet had no significant effect on the growth performance of broiler chickens (Hussein et al., 2019). There is limited research on the effects of reduced-protein or low-mineral diets with multi-enzyme addition on broilers.

The impact of adding enzyme preparations can differ based on the dietary ingredient composition, the enzyme profiles, and the addition methods (Zhang et al., 2018). The multi-enzyme preparation used in our experiment is a blend that consists of phytase, xylanase, protease, β-glucanase, cellulase, pectinase, and amylase. This product is beneficial to the growth of broilers. Furthermore, phytase releases bound phosphorus and calcium, improving bone mineralization and overall skeletal health (Humer et al., 2015). Hence, it is hypothesized that phytase supplementation will maintain bone mineralization despite the dietary reduction in both calcium and phosphorus. Moreover, information is lacking regarding the effects of multi-enzyme preparation with reduced levels of calcium and phosphorus in diets on the bone mineralization of broiler chickens. Therefore, this study aims to evaluate the effects of the multi-enzyme preparation (SSF) on the growth performance, bone mineralization, and intestinal development of broiler chickens fed a low-density diet. The findings may help formulate economical feeding approaches to improve poultry production efficiency and economic returns.

## Materials and methods

### Ethic Statement

All animal experiments were carried out in accordance with the Chinese Guidelines for Animal Welfare, following approval from the Ethics Committee of Qilu Normal University (2025QNU0211). Animal health and welfare were monitored at least twice daily throughout the trial. Handling and sampling were carried out under the supervision of registered veterinarians. The total mortality rate during the experiment was 12.5 %. In accordance with our experimental design and animal ethics guidelines, dead birds were promptly recorded and removed but were not replaced to maintain the integrity of the growth performance data within each replicate.

### Experimental animals and diets

**Fig. 1:**
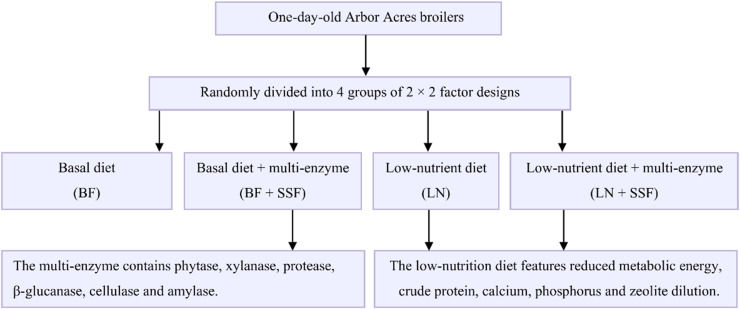
Research framework of the experimental design. A total of 384 one-day-old Arbor Acres broilers were randomly allocated to four dietary treatments: basal diet, basal diet with multi-enzymes, low-nutrient diet, and low-nutrient diet with multi-enzymes (*n* = 96).

A total of 384 one-day-old healthy Arbor Acres broilers with similar body weights were randomly divided into 4 groups in a 2 × 2 factorial arrangement, with 8 replicates per group and 12 chickens per replicate. The experimental diets consisted of a basal diet and a low-nutrient diet, each administered either with or without a multi-enzyme preparation (Fig. 1). This multi-enzyme preparation (Q/12ATQ 0044-2022, Tianjin Alltech Biological Products Co., Ltd.) contains phytase (≥ 300 U/g), xylanase (≥ 100 U/g), protease (≥ 700 U/g), β-glucanase (≥ 200 U/g), cellulase (≥ 40 U/g), and amylase (≥ 30 U/g). The basal diet was formulated to meet all nutrient requirements specified by the Chinese National Standard (GB/T 5916-2020). The low-nutrient diet was reduced in metabolizable energy by 150 kcal/kg, crude protein by 0.4 percentage units, calcium by 0.2 percentage units, and available phosphorus by 0.2 percentage units compared with the basal diet. The multi-enzyme preparation was supplemented at 200 mg/kg of diet, as recommended by the manufacturer. Thus, the experimental diets were divided into BF (basal diet group), BF+SSF (basal diet + multi-enzyme preparation group), LN (low-nutrient diet group), and LN+SSF (low-nutrient diet + multi-enzyme preparation group). Corn, soybean meal, corn gluten meal, corn distiller's grains, and soybean oil were used as the main raw materials. The feeding protocol of broilers was divided into two stages: starter (days 1–21) and finisher (days 22–35). The feeding and management of broilers referred to the methods of Cai et al. (2024). The broilers had access to feed and fresh water *ad libitum* throughout the experimental period. The experimental diets are shown in Table 1.

**Table 1:** Ingredients and composition of the experimental diets.

Items	Basal diet		Low-nutrient diet	
	day 1-21	day 22-35	day 1-21	day 22-35
Ingredients (%)				
Corn	57.70	59.71	51.51	53.58
Wheat	2.00	3.00	2.00	3.00
Distillers dried grains with solubles		2.50		2.50
Soybean oil	1.98	3.42	2.39	3.82
Soybean meal (46 % CP)	30.49	22.06	24.39	15.98
Peanut meal (50 % CP)	2.00	3.00	4.67	5.66
Corn gluten meal (60 % CP)	2.00	2.50	4.54	5.02
Sodium chloride	0.28	0.28	0.28	0.28
Limestone (37 %)	1.09	1.03	1.23	1.17
CaHPO_4_	1.39	1.20	0.25	0.05
Choline chloride (60 %)	0.10	0.10	0.10	0.10
L-Lysine (70 %)	0.38	0.57	0.50	0.69
DL-Methionine (99 %)	0.24	0.23	0.22	0.21
Threonine (98.5 %)	0.1	0.15	0.13	0.17
Zeolite power	0	0	7.54	7.52
Vitamin premix^a^	0.05	0.05	0.05	0.05
Trace element premix^b^	0.20	0.20	0.20	0.20
Total	100	100	100	100
Nutrient levels^c^				
Metabolizable energy (kcal/kg)	2870	2995	2720	2845
Crude protein (%)	21.45	19.45	21.05	19.05
Calcium (%)	0.80	0.72	0.60	0.52
Available phosphorus (%)	0.40	0.36	0.20	0.16
Lysine(%)	1.26	1.19	1.21	1.13
Methionine+cysteine(%)	0.93	0.87	0.89	0.83
Threonine (%)	0.88	0.83	0.85	0.80

a Vitamin premix (per kg of diet): vitamin A (retinylacetate), 10 000 IU; vitamin D_3_ (cholecalciferol), 2600 IU; vitamin E (DL-a-tocopherol acetate), 20 IU; vitamin K_3_ (menadione sodium bisulfate), 2 mg; vitamin B_1_ (thiamine mononitrate), 1.6 mg; vitamin B_2_ (riboflavin), 6 mg; vitamin B_6_ (pyridoxine hydrochloride), 3 mg; vitaminB_12_ (cyanocobalamin), 0.014 mg; nicotinic acid, 30 mg; pantothenic acid, 20 mg; biotin, 0.12 mg; folic acid, 0.8 mg; choline, 500 mg. The multi-enzyme supplement provided the following activities per kg of diet: phytase ≥ 60 U, xylanase ≥ 20 U, protease ≥ 140 U, β-glucanase ≥ 40 U, cellulase ≥ 8 U, and amylase ≥ 6 U.

b Mineral premix (per kg of diet): iron, 80 mg; copper, 8 mg; manganese, 100 mg; zinc, 80 mg; iodine, 0.35 mg; selenium, 0.15 mg.

c Calculated value.

### Growth performance

Body weight (BW) and feed consumption were recorded on days 1, 21, and 35 of the trial to determine the average daily feed intake (ADFI), BW, feed conversion ratio (FCR), and European performance index (EPI) for each period and cumulatively.

### Length and weight of each segment of the small intestine

At 21 and 35 days of age, one healthy broiler chicken from each replicate was selected and humanely euthanized via intravenous injection of sodium pentobarbital after 12 hours fasting. The small intestine was separated from the body. The duodenum (from the pylorus to the bile duct outlet), the jejunum (to the yolk sac remnant), and the ileum (to the ileocecal junction) were isolated. The length of each of the three intestinal segments was measured. The segments were then washed with distilled water, dried with filter paper, and weighed. The percentage of the absolute weight or length relative to body weight is referred to as the relative weight or length.

### Enzyme activity in duodenum mucosa

The duodenum mucosa was scraped with a sterile scalpel. For subsequent analysis, all tissue samples were frozen in liquid nitrogen and then transferred to −80°C for storage. The activities of lipase, trypsin, and amylase in the duodenum mucosa were measured using kits from the Nanjing Jiancheng Bioengineering Institute, China.

### Tibia indicators

The thigh muscle was removed, and the left tibia was collected for tibia length measurement. All soft tissues of the right tibiae were stripped after cooking at high temperature for 6 minutes, then dried at 105°C for 24 h, dehydrated, and soaked in 99.5 % pure ether for 96 h. The samples were then dried at 65°C for 4 h to constant weight, crushed through a 40-mesh sieve, mixed well, and ashed in a muffle furnace at 550°C for 24 h before being weighed. Ash content was calculated accordingly. The ash content was determined according to GB/T 6438-2007, the calcium content in ash was measured by the ethylene diamine tetraacetic acid (EDTA) complexometric titration method (GB/T 6436-2002), and the phosphorus content in ash was determined by the colorimetric method (GB/T 6437-2002).

### Statistical analysis

The main and interaction effects were analyzed using a two-factor analysis of variance in the general linear model of SPSS 26.0, with statistical significance set at *P* < 0.05. In cases where significant interaction effects were observed (P<0.05), Duncan’s multiple comparison test was used to further evaluate differences between groups. The results are expressed as mean ± standard error.

## Results

### Growth performance

The effects of dietary treatments on broiler growth performance are shown in Table 2. The BW and ADFI of broilers at 21 days of age were significantly reduced by feeding low-nutrient-level diets (*P* < 0.01). However, supplementation with the multi-enzyme preparation resulted in markedly higher BW and ADFI at 21 days of age in broilers fed the low-nutrient diet (*P* < 0.01). A significant interaction between dietary nutrient concentration and multi-enzyme addition was observed for BW and ADFI by day 21 (*P* < 0.01). At 35 days of age, diets with low nutrient levels reduced the BW and increased the feed conversion rate (FCR) of broilers (*P* < 0.01), while multi-enzyme addition improved the FCR and BW (*P* < 0.01). Furthermore, there was a significant interaction between nutrient level and multi-enzyme preparation with respect to BW and FCR (*P* < 0.05 and *P* < 0.01, respectively). In addition, the EPI of broilers throughout the entire trial was significantly reduced by feeding low-nutrient-level diets (*P* < 0.01), while multi-enzyme addition to the low-nutrient-level diet increased the EPI (*P* < 0.01).

**Table 2:** Effects of multi-enzyme preparation in low-nutrient diets on broiler growth performance.

Items	Treatment				SEM	*P* value		
	BF	BF+SSF	LN	LN+SSF		Nutrition	SSF	Nutrition × SSF
Day 1-21								
BW (g)	789.72^a^	832.05^a^	509.27^c^	731.75^b^	9.90	< 0.01	< 0.01	< 0.01
ADFI (g/d)	48.97^a^	50.79^a^	34.78^b^	46.95^a^	0.67	< 0.01	< 0.01	< 0.01
FCR	1.30	1.28	1.36	1.32	0.00	< 0.01	0.02	0.47
Day 22-35								
BW (g)	1865.16^ab^	1996.42^a^	1311.19^c^	1747.65^b^	32.12	< 0.01	< 0.01	0.03
ADFI (g/d)	131.46	139.84	102.54	128.34	3.00	< 0.01	< 0.01	0.16
FCR	2.06^b^	2.00^b^	2.47^a^	2.01^b^	0.03	< 0.01	< 0.01	< 0.01
Day 1-35								
ADFI (g/d)	89.46	93.90	91.46	86.70	2.75	0.64	0.98	0.41
FCR (%)	1.66	1.62	1.76	1.66	0.01	< 0.01	< 0.01	0.16
EPI	314.27^ab^	344.01^a^	124.32^c^	282.75^b^	8.18	< 0.01	< 0.01	<0.01

BW, body weight; ADFI, average daily feed intake; FCR, feed conversion ratio; EPI, European performance index; SEM, standard error of mean.

BF: basal diet group; BF+SSF: basal diet + multi-enzyme preparation group; LN: low nutrient diet group; LN+SSF: low nutrient diet + multi-enzyme preparation group.

A significance level of *P* < 0.05 was applied, and significant differences within the data are indicated by different superscript letters.

### Length and weight of each segment in the small intestine

Table 3 summarizes the influence of the multi-enzyme preparation in the reduced-nutrient diet on small intestinal morphology, including the length and weight of each segment at 21 and 35 days of age. At 21 days of age, the relative length of the duodenum, jejunum, and ileum of broilers was significantly increased by feeding low-nutrient-level diets (*P* < 0.01). Adding the multi-enzyme preparation to the low-nutrient diet significantly decreased the relative length of each segment of the small intestine (*P* < 0.01). At the same time, there was a significant interaction between nutrient level and multi-enzyme preparation regarding the relative length of the small intestine segments (*P* < 0.01). However, the relative weight of small intestinal segments remained unchanged across different dietary nutrient levels (*P* > 0.05). The experimental diets did not induce significant changes in either the relative dimensions or tissue weight of small intestinal segments at day 35 (*P* > 0.05).

**Table 3:** Effects of multi-enzyme preparation in low-nutrient diets on the length and weight of the small intestine segments.

Items	Group				SEM	*P* value		
	BF	BF+SSF	LN	LN+SSF		Nutrition	SSF	Nutrition × SSF
Day 21								
Relative length of the duodenum (cm/kg)	28.75^b^	27.43^b^	39.24^a^	29.60^b^	0.41	< 0.01	< 0.01	< 0.01
Relative weight of the duodenum (g/kg)	7.51	7.13	7.85	6.88	0.13	0.87	0.01	0.27
Relative length of the jejunum (cm/kg)	65.19^b^	59.77^b^	88.81^a^	63.38^b^	0.95	< 0.01	< 0.01	< 0.01
Relative weight of the jejunum(g/kg)	13.22	11.73	12.29	11.09	0.22	0.08	<0.01	0.75
Relative length of the ileum (cm/kg)	63.43^b^	58.44^b^	88.59^a^	63.57^b^	1.28	< 0.01	< 0.01	< 0.01
Relative weight of the ileum(g/kg)	10.68	9.69	10.43	9.53	0.16	0.53	< 0.01	0.89
Day 35								
Relative length of the duodenum (cm/kg)	16.20	15.42	17.47	16.44	0.51	0.27	0.38	0.90
Relative weight of the duodenum (g/kg)	5.36	6.01	5.77	5.25	0.22	0.70	0.88	0.20
Relative length of the jejunum (cm/kg)	35.89	35.47	38.27	36.49	1.21	0.49	0.65	0.78
Relative weight of the jejunum(g/kg)	10.29	11.65	10.01	10.10	0.30	0.14	0.24	0.30
Relative length of the ileum (cm/kg)	35.11	38.73	40.22	37.75	1.16	0.38	0.81	0.20
Relative weight of the ileum(g/kg)	7.59	7.95	8.80	8.63	0.27	0.09	0.86	0.63

BF: basal diet group; BF+SSF: basal diet + multi-enzyme preparation group; LN: low nutrient diet group; LN+SSF: low nutrient diet + multi-enzyme preparation group. SEM, standard error of mean.

A significance level of *P* < 0.05 was applied, and significant differences within the data are indicated by different superscript letters.

### Activities of digestive enzymes

The effects of multi-enzyme preparation supplementation in the low-nutrient diet on the activities of digestive enzymes in the duodenal mucosa are shown in Table 4. At 21 days of age, diets with low nutrient levels reduced the activity of lipase in the duodenal mucosa (*P* < 0.01), while multi-enzyme supplementation significantly increased the amylase activity in the duodenal mucosa (*P* < 0.01). At 35 days of age, the trypsin activity in the duodenal mucosa decreased in response to the low-nutrient diet (*P* < 0.01). However, the lipase activity increased in broilers fed the low-nutrient diet (*P* < 0.01). A significant interaction effect between low-nutrient diets and multi-enzyme addition was observed for amylase activity (*P* < 0.05).

**Table 4:** Effects of multi-enzyme preparation in low-nutrient diets on activity of digestive enzymes in duodenal mucosa.

Parameters	Group				SEM	*P* value		
	BF	BF+SSF	LN	LN+SSF		Nutrition	SSF	Nutrition × SSF
Day 21								
Trypsin (U/mg prot)	33.27	38.65	38.28	38.45	1.70	0.49	0.42	0.45
Lipase (U/mg prot)	21.66	26.73	11.38	10.06	1.74	< 0.01	0.59	0.37
Amylase (U/mg prot)	0.41	0.50	0.43	0.49	0.01	0.76	<0.01	0.56
Day 35								
Trypsin (U/mg prot)	67.93	79.30	61.64	59.69	1.87	< 0.01	0.22	0.09
Lipase (U/mg prot)	17.35	21.58	29.18	28.20	1.01	< 0.01	0.43	0.21
Amylase (U/mg prot)	0.57^b^	0.67^ab^	0.76^a^	0.60^ab^	0.03	0.30	0.59	0.03

BF: basal diet group; BF+SSF: basal diet + multi-enzyme preparation group; LN: low nutrient diet group; LN+SSF: low nutrient diet + multi-enzyme preparation group. SEM, standard error of mean.

A significance level of *P* < 0.05 was applied, and significant differences within the data are indicated by different superscript letters.

### Tibial parameters

The effects of the multi-enzyme preparation in low-nutrient diets on tibial length and tibial calcium and phosphorus contents are presented in Table 5. Reduction of dietary nutrient levels led to a shorter tibial length in 21-day-old broilers (*P* < 0.01). However, adding the multi-enzyme preparation to the low-nutrient diet significantly increased the tibial length (*P* < 0.01). Furthermore, a significant interaction between nutrient level and the multi-enzyme mixture was observed regarding tibial length at day 35 (*P* < 0.05). At 21 days of age, diets with low nutrient levels reduced the calcium content in the tibia (*P* < 0.05), while the multi-enzyme preparation in the low-nutrient diet significantly increased the calcium content (*P* < 0.01). Unexpectedly, broilers fed the low-nutrient diet showed higher phosphorus content in the tibia at day 21 (*P* < 0.01). In addition, calcium and phosphorus contents in the tibia were not affected by dietary factors at 35 days of age (*P* > 0.05).

**Table 5:** Effects of multi-enzyme preparation in low-nutrient diets on tibial parameters.

Parameters	treatment				SEM	*P* value		
	BF	BF+SSF	LN	LN+SSF		Nutrition	SSF	Nutrition × SSF
Tibial length (cm)								
day 21	6.71	7.08	5.91	6.49	0.04	< 0.01	<0.01	0.21
day 35	8.95^a^	8.85^ab^	8.56^b^	8.99^a^	0.05	0.25	0.13	0.02
Calcium content in Tibia (%)								
day 21	18.57	23.75	16.83	20.19	0.56	0.04	<0.01	0.43
day 35	32.19	31.58	36.46	36.41	1.32	0.11	0.90	0.92
Phosphorus content in tibia (%)								
day 21	9.55	9.51	9.72	9.76	0.03	< 0.01	0.98	0.54
day 35	15.19	14.85	16.12	16.58	0.45	0.16	0.95	0.66

BF, basal diet group; BF+SSF, basal diet + multi-enzyme preparation; LN, low nutrient diet group; LN+SSF, low nutrient diet + multi-enzyme preparation group; SEM: standard error of the mean.

A significance level of *P* < 0.05 was applied, and significant differences within the data are indicated by different superscript letters.

## Discussion

### Growth performance

Feed expenses constitute approximately 60–75 % of total poultry production costs, making efficient utilization of feed components and additives crucial for optimizing poultry growth outcomes. Low-nutrient feeds may save costs but negatively impact production performance. While poultry naturally synthesize certain digestive enzymes, approximately 25 % of dietary components remain undigested because of inherent anti-nutritional factors in feed ingredients (Bedford and Partridge, 2010). However, multi-enzyme supplementation enhances growth performance by boosting nutrient absorption efficiency (Sharifi et al., 2013). The inclusion of a multi-enzyme preparation (amylase, xylanase, and protease) enhanced energy utilization in both corn- and sorghum-soybean meal diets. This effect is attributed to the targeted breakdown of starch, cell wall components, and endogenous proteins by the respective enzymes (He et al., 2022). Therefore, the strategic incorporation of multi-enzyme blends into reduced-nutrient diets represents a potential nutritional intervention strategy without compromising growth performance. In the present study, the low-nutrient diet decreased BW on day 21 and day 35, increased FCR during the finisher stage, and lowered the EPI throughout the entire experimental period. However, multi-enzyme supplementation in the low-nutrient diet improved the above growth parameters, implying that multi-enzyme supplementation can improve growth performance and correct the negative effects of the reduced nutrient level. Zeolite was chosen primarily for its inert properties and high nutrient-binding capacity, making it a suitable diluent to physically reduce the nutrient density of the feed without introducing other active components. A substantial body of research (Emam et al., 2019; Pavlak et al., 2022; AL-Musawy et al., 2023, 2023) has consistently reported that dietary zeolite supplementation, at appropriate levels, exerts a growth-promoting or neutral effect in poultry. It was highly unlikely that it would cause the growth depression we observed. Therefore, the significant performance reduction in the LN group was more plausibly attributed to the nutrient dilution itself. Furthermore, since zeolite was present in both the LN and LN+SSF diets, any positive effect originating from zeolite itself would be a constant factor in both. The significant performance recovery observed in the LN+SSF group compared to the LN group could, therefore, be more confidently attributed to the action of the enzyme preparation, which compensated for the nutrient dilution. These findings are in line with those reported by Attia et al. (2022), who found that a multi-enzyme mixture enhanced growth performance and economic traits, especially in low-nutrient diets for broiler chickens.

In our current study, the enhanced growth performance observed with low-nutrient diets supplemented with multi-enzymes likely stems from the combined action of protease, amylase, and NSP-degrading enzymes. The inclusion of exogenous NSP-degrading enzymes in vegetable-derived feeds led to marked reductions in digesta viscosity and concomitant enhancements in energy availability and nutrient digestibility (Ayalew et al., 2022). In addition, some starch granules are encapsulated within a protein matrix and are not easily accessible to amylase (Zaefarian et al., 2015). In cereal grains, protease supplementation could promote the breakdown of protein matrices associated with NSP and starch, leading to better nutrient absorption. Moreover, according to Kim et al. (2021), the enhanced villus height appeared to result primarily from the synergistic action of the multi-enzyme mixture. The improvement of intestinal morphology promotes the absorption of nutrients, thereby enhancing production performance. Furthermore, Olukosi and Adeola (2008) reported that a complex of xylanase, amylase, protease, and phytase synergistically improved growth performance in diets deficient in ME and phosphorus. Therefore, our results suggest that the growth enhancement was principally mediated by synergistic interactions among the enzyme components.

### Small intestinal weight and length

The low-nutrient diets in the present study increased the length percentages of the duodenum, jejunum, and ileum at day 21, indicating an adaptive response in the gut. Consistent with the findings of Attia et al. (2022) on low-density diets, a low-nutrient diet induces a compensatory response in gut morphology, notably intestinal elongation, to expand the absorptive surface. This adaptation slows chyme passage, allowing for prolonged contact with digestive juices and thus improved nutrient breakdown and feed efficiency. This represents a compensatory response of the body to the reduction of nutrients. However, the relative weight of intestinal segments was not affected by the low-nutrient diets. In agreement with our findings, Irmak et al. (2024) reported that total intestine weights were not affected by multi-enzyme supplementation. The reason might be that the muscle layer of the small intestine became thinner. In addition, by day 35, nutrient levels and multi-enzymes did not influence the relative length and weight of intestinal segments in chickens, implying increased tolerance to the diets as broilers aged.

During the starter phase, the inclusion of the multi-enzyme in low-nutrient diets resulted in a reduction in the length of all small intestinal segments (duodenum, jejunum, and ileum). Similarly, Wu et al. (2004) demonstrated that enzyme inclusion in the diet decreased the relative length of the small intestine in broilers. The weight and length of gastrointestinal organs may vary in response to alterations in feed intake levels, dietary formulations, or nutrient concentration. Grains containing higher levels of NSP may promote gastrointestinal tract enlargement (Asare et al., 2022). The multi-enzyme preparation used in our trial contains NSP-degrading enzymes, such as xylanase, β-glucanase, cellulase, and pectinase, which can reduce the NSP content in the intestine, leading to decreased intestinal length. In addition, enzyme supplementation in corn-soybean meal diets may break down NSP, potentially attenuating the secretion of digestive organs and contributing to reduced digestive organ size (Wu et al., 2004).

### Activities of digestive enzymes

Broiler chickens inherently digest starch well, but their efficiency can be reduced by factors such as low endogenous amylase production and the physical nature of starch granules. Amylase supplementation enhances broiler growth performance, feed efficiency, and body weight gain by improving the utilization of dietary carbohydrates (Ibrahim et al., 2023). Improved growth performance was observed in the present study, which was mediated by the enhanced duodenal mucosal amylase activity on day 21 following supplementation with an amylase-containing multi-enzyme preparation. This finding is consistent with a previous study in which amylase inclusion improved the overall BW gain and FCR of chickens at 21 days of age (Aderibigbe et al., 2020). Therefore, the growth performance improvements observed in the broilers could be partly attributed to exogenous amylase addition. However, the addition of the multi-enzyme preparation to the diet did not influence the activities of trypsin, lipase, or amylase on day 35. The effects of exogenous enzymes on digestive enzyme activities have been reported inconsistently. Shakouri et al. (2009) reported that enzyme supplementation did not influence intestinal enzyme activity. In contrast, supplementing a low-ME diet with multi-enzymes elevated lipase and protease activities in the jejunal digesta, while amylase activity remained unchanged (Yaqoob et al., 2022). The digestive function of the body depends on the volume of digestive juice secreted by the liver, pancreas, and intestinal mucosa, as well as the activities of enzymes. Unfortunately, only the digestive enzyme activity in the duodenal mucosa was measured in our trial, which is insufficient to fully reflect the digestive capacity of the broilers.

### Tibial development

Leg abnormalities in broiler chickens represent significant challenges for both animal welfare and economic efficiency. Various factors, including genetics, dietary imbalances, and rapid growth rates, can contribute to skeletal deformities in broilers, resulting in reduced feed intake and impaired weight gain (Khaleghi et al., 2025). Thus, dietary adjustments could help mitigate leg abnormalities in broilers (Williams et al., 2000). A reduced-nutrient-density diet could serve as a viable strategy to limit feed costs and reduce the growth-related load on the skeletal system of broilers (Attia, 2001).

In the present study, the low-nutrient diet decreased the length and calcium content of the tibia at day 21, implying that a reduction in calcium and total phosphorus by 0.2 percentage points was sufficient to affect bone development in broilers. However, multi-enzyme supplementation can mitigate the negative effects of low-nutrient diets. Similarly, enzyme supplementation in a low-phosphorus diet improved tibia mineralization (Walters et al., 2019).

Phytate-bound phosphorus represents 60–80 % of the total phosphorus content in corn- and soybean meal-based diets. Phytate's anti-nutritional effects stem from its ability to bind minerals and nutrients into indigestible complexes while increasing metabolic waste, ultimately reducing digestion efficiency and impairing poultry productivity (Woyengo and Nyachoti, 2013). Dietary enzyme supplementation promotes bone mineralization in broilers by increasing the bioavailability of essential nutrients from feed ingredients (Abu-Tayyeb et al., 2019). Specifically, the inclusion of NSP-degrading enzymes decreases digesta viscosity, enhances nutrient uptake, and contributes to improved bone strength and mineralization (Bauer, 2023).

Notably, a synergistic interaction has been observed between phytase and NSP-degrading enzymes. Multi-enzymes and phytase synergistically improved the digestibility of dry matter, crude protein, calcium, and phosphorus (Kim et al., 2021). According to Lu et al. (2013), supplementation with a xylanase, β-glucanase, and phytase complex elevated the dietary utilization of dry matter, crude protein, calcium, and phosphorus in reduced-nutrient diets for chickens. This improved digestibility likely underlies the enhanced tibia development observed with enzyme supplementation. A surprising finding in our study was that broilers receiving the low-nutrient diet exhibited lower tibia calcium content but higher phosphorus content at day 21. This result is biologically unexpected and the mechanisms behind this phenomenon warrant further investigation.

## Conclusion

**Fig. 2:**
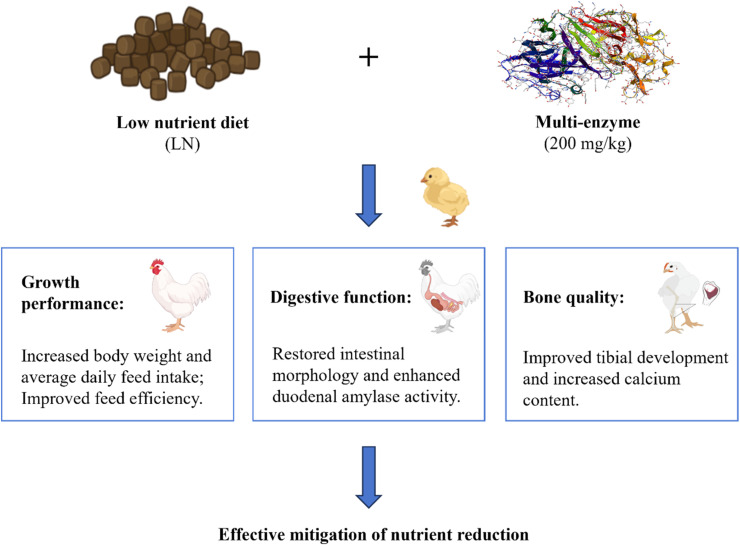
Graphical summary of the key findings. Multi-enzyme supplementation mitigates the adverse effects of a zeolite-diluted, low-nutrient diet on growth performance, digestive function and bone quality in broilers.

This study shows that multi-enzyme supplementation acts as an effective strategy to combat the negative impacts of zeolite-diluted, low-nutrient diets in broilers (Fig. 2). The improvements in growth performance and tibial development, along with enhanced duodenal amylase activity, indicate that the enzymes' function extends beyond nutrient release to modulating systemic metabolism and skeletal health. While this study underscores the potential of this nutritional strategy, future research should elucidate the underlying mechanisms (particularly at molecular, histological, and biochemical levels) and validate these findings under diverse commercial settings.

## CRediT authorship contribution statement

**Yuanli Cai:** Writing – original draft, Methodology. **Mingchen Zhang:** Project administration, Data curation. **Okasha Hamada:** Writing – review & editing. **Zhigang Song:** Writing – review & editing, Funding acquisition, Conceptualization.

## Disclosures

The authors declare that they have no known competing financial interests or personal relationships that could have appeared to influence the work reported in this paper.
